# Highly Sensitive Room‐Temperature Detection of Ammonia in the Breath of Kidney Disease Patients Using Fe_2_Mo_3_O_8_/MoO_2_@MoS_2_ Nanocomposite Gas Sensor

**DOI:** 10.1002/advs.202405942

**Published:** 2024-07-03

**Authors:** Xian Li, Wang Zeng, Shangjun Zhuo, Bangwei Qian, Qiao Chen, Qun Luo, Rong Qian

**Affiliations:** ^1^ National Centre for Inorganic Mass Spectrometry in Shanghai Shanghai Institute of Ceramics Chinese Academy of Sciences Shanghai 200050 P. R. China; ^2^ Centre of Materials Science and Optoelectronics Engineering University of Chinese Academy of Sciences Beijing 100864 P. R. China; ^3^ School of Material Science and Engineering Shanghai University Shanghai 200444 P. R. China; ^4^ Shanghai Pudong New Area People's Hospital Shanghai 201299 P. R. China; ^5^ Department of Chemistry School of Life Sciences University of Sussex Brighton BN1 9QJ UK

**Keywords:** ammonia, exhaled breath, Fe_2_Mo_3_O_8_/MoO_2_@MoS_2_ nanocomposite, kidney disease, room‐temperature

## Abstract

A novel Fe_2_Mo_3_O_8_/MoO_2_@MoS_2_ nanocomposite is synthesized for extremely sensitive detection of NH_3_ in the breath of kidney disease patients at room temperature. Compared to MoS_2_, α‐Fe_2_O_3_/MoS_2_, and MoO_2_@MoS_2_, it shows the optimal gas‐sensing performance by optimizing the formation of Fe_2_Mo_3_O_8_ at 900 °C. The annealed Fe_2_Mo_3_O_8_/MoO_2_@MoS_2_ nanocomposite (Fe_2_Mo_3_O_8_/MoO_2_@MoS_2_‐900 °C) sensor demonstrates a remarkably high selectivity of NH_3_ with a response of 875% to 30 ppm NH_3_ and an ultralow detection limit of 3.7 ppb. This sensor demonstrates excellent linearity, repeatability, and long‐term stability. Furthermore, it effectively differentiates between patients at varying stages of kidney disease through quantitative NH_3_ measurements. The sensing mechanism is elucidated through the analysis of alterations in X‐ray photoelectron spectroscopy (XPS) signals, which is supported by density functional theory (DFT) calculations illustrating the NH_3_ adsorption and oxidation pathways and their effects on charge transfer, resulting in the conductivity change as the sensing signal. The excellent performance is mainly attributed to the heterojunction among MoS_2_, MoO_2_, and Fe_2_Mo_3_O_8_ and the exceptional adsorption and catalytic activity of Fe_2_Mo_3_O_8_/MoO_2_@MoS_2_‐900 °C for NH_3_. This research presents a promising new material optimized for detecting NH_3_ in exhaled breath and a new strategy for the early diagnosis and management of kidney disease.

## Graphical abstract

An ultrasensitive NH_3_ gas sensor is developed based on Fe_2_Mo_3_O_8_/MoO_2_@MoS_2_‐900 °C nanocomposite. The sensor effectively distinguishes between patients with early‐ and late‐stage kidney disease by quantitative analysis of NH_3_ in their exhaled breath. This novel approach offers a promising method for the early diagnosis and management of kidney disease.

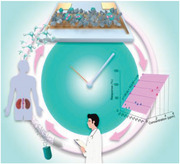

## 1 Introduction

Ammonia (NH_3_) is a key component in amino acids and proteins. It originates from a variety of sources, such as animal and plant substances, organic decomposition, and industrial wastewater.^[^ ^1^ ^]^ Although NH_3_ can negatively affect the environment and human health, its detection has many practical applications. In particular, it can be used as a breath signature for early screening or indirect diagnosis of some diseases. Poor liver function can delay the metabolism of NH_3_, while impaired kidney function can delay the excretion of metabolic end products such as urea. Patients with such disease could have a high level of NH_3_, detectable in their breath.

Conventional methods of diagnosing kidney disease, such as blood measurements, urine tests, renal biopsies, B‐ultrasounds, computed tomography scans, or magnetic resonance imaging, are typically invasive, uncomfortable, time‐consuming, and costly. Moreover, these methods are only suitable for a limited range of patients. Hence, the use of NH_3_ as a breath signature offers a convenient, low‐cost method for the early screening and diagnosis of kidney disease, even in a domestic environment. Previous studies have reported that NH_3_ concentrations of >10 ppb in the breath of a patient may indicate liver or kidney failure.^[^ ^2^ ^]^ Therefore, it is a great challenge to develop a cost‐effective and portable but still highly sensitive and selective NH_3_ sensor with a detection limit better than 10 ppb. Such devices can also be adapted for industrial applications such as environmental protection, public safety, and chemical leakage monitoring.

A variety of techniques have been used to detect and monitor NH_3_, including optical, chromatographic, spectroscopic, and mass spectrometric techniques.^[^ ^3^ ^]^ Nevertheless, these methods require complex pretreatments, bulky equipment, and intricate processes at high costs, which have hindered their widespread application.^[^ ^4^, ^5^ ^]^ Gas sensors, particularly metal‐oxide‐semiconductor sensors, could offer sensitive, portable, user‐friendly, and cost‐effective NH_3_ detection, making them highly suitable for noninvasive screening applications. Nguyen et al. sputter deposited SnO_2_/Pt/WO_3_ ternary films on silicon oxide substrates, which achieved a response of 46.3 for 100 ppm of NH_3_ at 250 °C.^[^ ^6^ ^]^ Kan et al. constructed a dual‐mode foam sensor based on Ti_3_C_2_T_x_/In_2_O_3_ nanocomposite for noninvasive detection of kidney disease by measuring the NH_3_ concentration. The results showed that the resistance of the sensor changed by at least 0.1 MΩ in a simulated exhaled breath test with a detection limit of 1 ppm at room temperature.^[^ ^7^ ^]^ Wang et al. developed a wearable respiratory sensor using CeO_2_@polyaniline nanocomposite to detect trace levels of NH_3_ with a relatively low response of 300% for 30 ppm NH_3_.^[^ ^8^ ^]^ Zhao et al. proposed a proton‐conductive gas sensor based on polyvinylpyrrolidone to detect 0.5 ppm NH_3_ of exhaled breath in simulated environments with an estimated detection limit of 36 ppb.^[^ ^9^ ^]^ Thus, the difficulty remains in developing highly sensitive NH_3_ detectors that are capable of measuring the NH_3_ concentration as low as 10 ppb in order to provide early diagnosis of kidney diseases before the presence of other typical symptoms. Hence it is urgently needed to develop new material that is sensitive enough to detect NH_3_ with good selectivity.

MoS_2_ has been identified as a promising candidate for detecting molecules containing nitrogen due to its strong interaction between Mo and N.^[^ ^10^ ^]^ Zhang et al. fabricated MoS_2_/Co_3_O_4_ thin film sensors on interdigitated electrode substrates with 62.4% response to 5 ppm NH_3_ and fast response/recovery for NH_3_ detection.^[^ ^11^ ^]^ Wang et al. prepared a SnO_2_/MoS_2_ nanocomposite, which showed a good response of 2080.36 for 200 ppm of NH_3_ and a fast response/recovery time of 23/1.6 s for 50 ppm of NH_3_ at 25 °C.^[^ ^12^ ^]^ However, the detection of NH_3_ in the exhaled breath for patients with kidney disease using gas sensors remains limited due to the ultra‐low NH_3_ concentration and potential interference with other gases, including moisture. As a result, most of the current gas sensors only offer qualitative detection of ammonia gas in exhaled breath, which provides less accurate and meaningful information. The difficulty in detecting low NH_3_ concentration for early diagnosis needs to be addressed, which is essential for the early treatment of kidney disease.

In this study, an ultrasensitive NH_3_ gas sensor was constructed using a Fe_2_Mo_3_O_8_/MoO_2_@MoS_2_ nanocomposite. The optimal gas‐sensing performance was achieved by optimizing the formation Fe_2_Mo_3_O_8_ at 900 °C under a low oxygen partial pressure. This optimal sample is named Fe_2_Mo_3_O_8_/MoO_2_@MoS_2_‐900 °C, which shows a high response of 875% for 30 ppm of NH_3_, with an ultralow detection limit of 3.7 ppb, when operating at room temperature. It also shows excellent selectivity for NH_3_ with long‐term stability observed in a four‐week test. Such a superior detection limit for NH_3_ is critical for the application in early diagnosis of kidney disease.

Moreover, our Fe_2_Mo_3_O_8_/MoO_2_@MoS_2_‐900 °C sensor was successfully employed to differentiate patients with early‐ and late‐stage kidney disease by quantitative analysis of NH_3_ in the exhaled breath. This confirms that our sensor is sensitive enough to identify the early stage of kidney disease with good reliability, providing the opportunity for early medical intervention. The sensing mechanism was elucidated through the analysis of the interaction between Fe_2_Mo_3_O_8_ and NH_3_, the adsorption and oxidation of NH_3_, and the charge transfer mechanism, which is manifested through the drastic change of the conductivity upon exposure to NH_3_. This was supported by Ab Initio density functional theory (DFT) calculations of the adsorption energy of NH_3_ on sensing materials and the free energy changes during NH_3_ oxidation. This work provides a new material that offers ultra‐sensitivity to NH_3_ detection in the exhaled breath of patients with kidney disease. The application of this technology can potentially provide a useful domestic medical device for early diagnosis of kidney functioning.

## 2 Results and Discussion

### 2.1 Material Characterization

**Figure 1:**
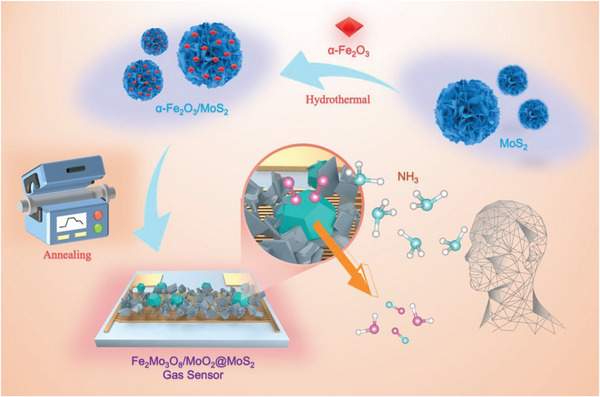
Schematic diagram of the synthesis of Fe_2_Mo_3_O_8_/MoO_2_@MoS_2_‐900 °C nanocomposite and the fabrication of Fe_2_Mo_3_O_8_/MoO_2_@MoS_2_‐900 °C gas sensor.

The Fe_2_Mo_3_O_8_/MoO_2_@MoS_2_‐900 °C nanocomposite was prepared through a secondary hydrothermal method and an annealing process and subsequently utilized for the fabrication of an NH_3_ gas sensor, as illustrated in **Figure** **1**.

**Figure 2:**
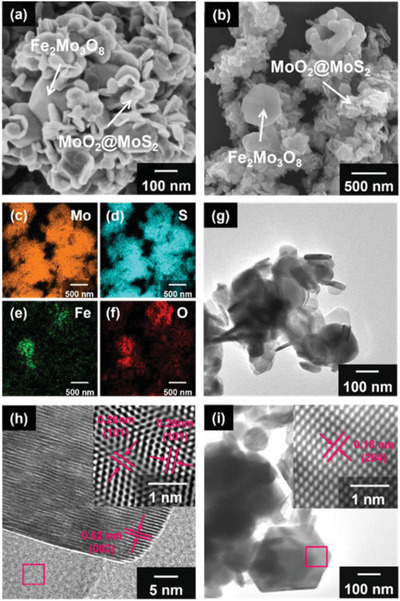
a) SEM image and b) EDS mapping of Fe_2_Mo_3_O_8_/MoO_2_@MoS_2_‐900 °C, c–f) Elemental maps of Mo, S, Fe, and O in Fe_2_Mo_3_O_8_/MoO_2_@MoS_2_‐900 °C. g) TEM image of Fe_2_Mo_3_O_8_/MoO_2_@MoS_2_‐900 °C. HR‐TEM images of h) MoO_2_@MoS_2_ and i) Fe_2_Mo_3_O_8_ in Fe_2_Mo_3_O_8_/MoO_2_@MoS_2_‐900 °C.

Field‐emission scanning electron microscopy (SEM) and high‐resolution transmission electron microscopy (HR‐TEM) were employed to examine the surface morphology and lattice structure of the as‐prepared Fe_2_Mo_3_O_8_/MoO_2_@MoS_2_‐900 °C nanocomposite. In **Figure** **2**a, the SEM image shows the Fe_2_Mo_3_O_8_/MoO_2_@MoS_2_‐900 °C nanocomposite, revealing MoO_2_@MoS_2_ disks with an average diameter of ≈100 nm. Within these disks, Fe_2_Mo_3_O_8_ particles with regular polyhedral structures and an average diameter of 300 nm could be observed. Figure 2b presents the SEM energy dispersive spectrometer (EDS) mapping of the Fe_2_Mo_3_O_8_/MoO_2_@MoS_2_‐900 °C nanocomposite, clearly distinguishing the Fe_2_Mo_3_O_8_ and MoO_2_@MoS_2_ compounds based on their distinct morphologies. Elemental maps of Mo, S, Fe, and O (Figure 2c–f) corresponded to the regions identified in Figure 2b. The Mo and S elements were evenly distributed throughout the composite, indicating the predominance of MoS_2_. Fe and O elements were concentrated at the large particles Fe_2_Mo_3_O_8_. Additionally, a small amount of O was observed outside of the Fe_2_Mo_3_O_8_ particles, which could be attributed to the substitution of S by O in the MoS_2_ structure, resulting in the formation of the MoO_2_@MoS_2_ phase. SEM and EDS mapping images proved the formation of Fe_2_Mo_3_O_8_/MoO_2_@MoS_2_ ternary heterojunction, which could enhance the material gas‐sensing performance.

The TEM image of the as‐prepared Fe_2_Mo_3_O_8_/MoO_2_@MoS_2_‐900 °C nanocomposite is shown in Figure 2g. The lattice structure of MoO_2_@MoS_2_ was observed in Figure 2h, with lattice stripe spacing of 0.62, 0.26, and 0.28 nm corresponding to (002), and (101) planes of MoS_2_, and (101) plane of MoO_2_, respectively. The presence of alternating S and O atoms in MoO_2_@MoS_2_ resulted in lattice stress and significant lattice distortion, which in turn provided a large number of active sites for gas‐sensing response. Figure 2i shows the lattice structure of the Fe_2_Mo_3_O_8_, revealing a lattice stripe spacing of 0.18 nm corresponding to the (204) plane. This observation confirms the presence of Fe_2_Mo_3_O_8_ in the Fe_2_Mo_3_O_8_/MoO_2_@MoS_2_‐900 °C nanocomposite.

**Figure 3:**
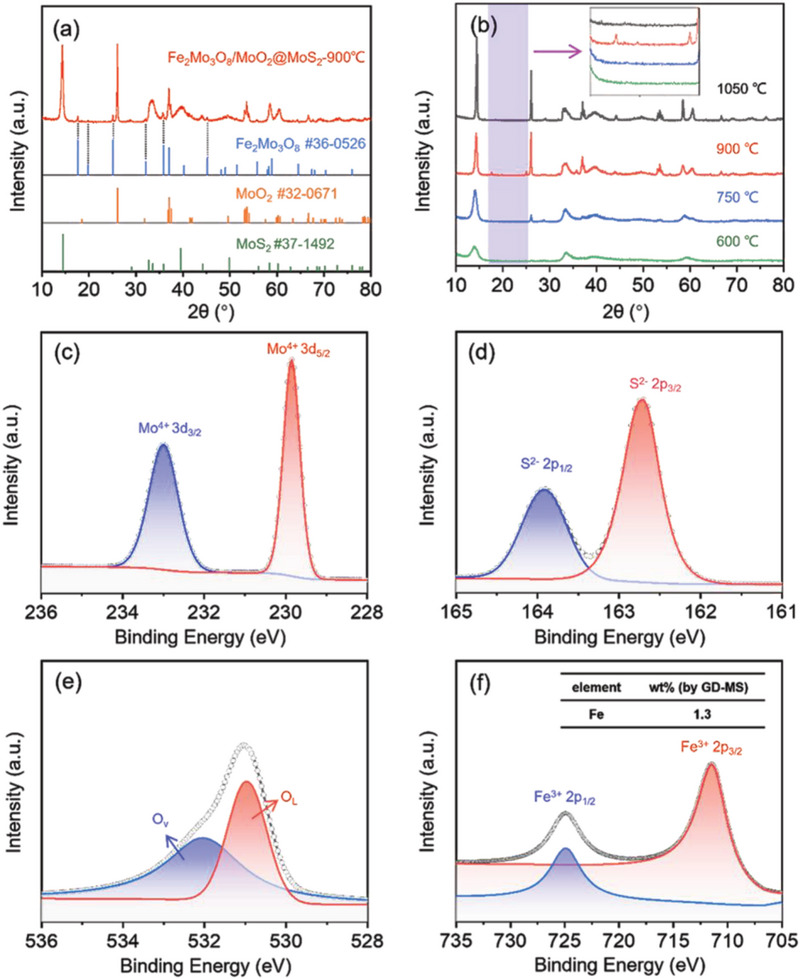
XRD spectra of a,b) Fe_2_Mo_3_O_8_/MoO_2_@MoS_2_‐900, FeMoOS‐600, FeMoOS‐750, and FeMoOS‐1050 °C. XPS spectra of c) Mo, d) S, e) O, and f) Fe peaks.

X‐ray diffraction (XRD) was used to analyze the structures of α‐Fe_2_O_3_/MoS_2_ nanocomposite annealed at 600, 750, 900, and 1050 °C (labeled FeMoOS‐600, FeMoOS‐750, Fe_2_Mo_3_O_8_/MoO_2_@MoS_2_‐900, and FeMoOS‐1050 °C) respectively. The XRD from the Fe_2_Mo_3_O_8_/MoO_2_@MoS_2_‐900 °C composite is shown in **Figure** **3**a. The diffraction peaks at 17.6, 25.1, and 35.9° are assigned to the (002), (102), and (112) crystal planes of the Fe_2_Mo_3_O_8_. The diffraction peaks at 26.0, 36.8, 36.9, 37.0, 37.4, and 49.5° were assigned to the (−111), (200), (111), (−211), (−202), and (−301) crystal planes of monoclinic MoO_2_. The diffraction peaks at 14.4, 32.7, 39.5, 49.8, and 58.3 were assigned to the (002), (100), (103), (105), and (110) crystal planes of MoS_2_. Comparing the peak intensities and positions of the samples indicated that the nanocomposite was composed of Fe_2_Mo_3_O_8_, MoO_2_, and MoS_2_. Figure 3b illustrates the evolution of the nanocomposites annealed at different temperatures. It was obvious that MoO_2_ was formed in FeMoOS‐750, FeMoOS‐1050, and Fe_2_Mo_3_O_8_/MoO_2_@MoS_2_‐900 °C, while Fe_2_Mo_3_O_8_/MoO_2_ only appeared in Fe_2_Mo_3_O_8_/MoO_2_@MoS_2_‐900 °C, identified by the diffraction peaks at 17.6 and 25.1°. Based on XRD quantitative analysis, the mass percentages of Fe_2_Mo_3_O_8_, MoO_2_, and MoS_2_ in Fe_2_Mo_3_O_8_/MoO_2_@MoS_2_‐900 °C are 1.0%, 6.3%, and 92.7%, respectively.

X‐ray photoelectron spectroscopy (XPS) was also used to determine the surface composition and chemical state of the Fe_2_Mo_3_O_8_/MoO_2_@MoS_2_‐900 °C nanocomposite, as shown in Figure 3c–f. There were two peaks in the Mo 3d spectrum at 229.85 and 232.97 eV, corresponding to the Mo^4+^ d_5/2_ and Mo^4+^ d_3/2_ in MoS_2_ and MoO_2_, respectively. There were two peaks in the S 2p spectrum at 162.73 and 163.89 eV, assigned to the S^2−^ 2p_3/2_ and S^2−^ 2p_1/2_, respectively. There were two peaks in the Fe 2p spectrum at 711.48 and 724.92 eV, corresponding to Fe^3+^ 2p_3/2_ and Fe^3+^ 2p_1/2_, respectively. Finally, there were two peaks at 530.93 and 532.05 eV, which corresponded to lattice oxygen (O_L_) and oxygen vacancies (O_V_), respectively. The oxygen vacancies provide active sites for gas adsorption. When the Fe_2_Mo_3_O_8_/MoO_2_@MoS_2_‐900 °C sensor was exposed to air, the oxygen vacancies might facilitate the activation of adsorbed O_2_, forming $O_{2}^{-}.$ Upon exposure to NH_3_ gas, the $O_{2}^{-}$ will oxidize the adsorbed surface NH_3_. Thus, the combination of oxygen vacancies and lattice oxygen stability is crucial for optimizing the NH_3_ gas‐sensing performance.^[^ ^13^ ^]^

### 2.2 Gas‐Sensing Properties

#### 2.2.1 Effect of Annealing Temperatures on Gas‐Sensing Performance

**Figure 4:**
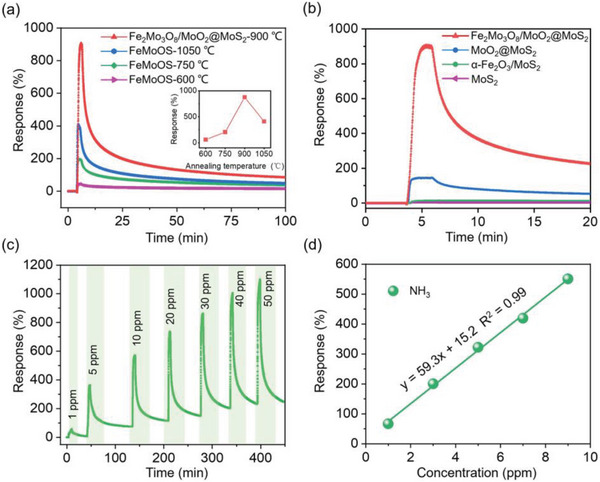
a) Gas‐sensing responses of FeMoOS‐600, FeMoOS‐750, Fe_2_Mo_3_O_8_/MoO_2_@MoS_2_‐900, and FeMoOS‐1050 °C toward 30 ppm of NH_3_ at room temperature and 5% RH. b) Gas‐sensing responses of MoS_2_, α‐Fe_2_O_3_/MoS_2_, MoO_2_@MoS_2_ and Fe_2_Mo_3_O_8_/MoO_2_@MoS_2_‐900 °C to 30 ppm of NH_3_ at room temperature and 5% RH. c) Sensitivity of Fe_2_Mo_3_O_8_/MoO_2_@MoS_2_‐900 °C for NH_3_ at concentrations of 1–50 ppm at room temperature and 5% RH. d) The calibration curve between NH_3_ concentration and response for Fe_2_Mo_3_O_8_/MoO_2_@MoS_2_‐900 °C.

To investigate the effect of annealing temperatures ranging between 600 to 1050 °C on the NH_3_ gas‐sensing performance, four nanocomposites, namely FeMoOS‐600, FeMoOS‐750, Fe_2_Mo_3_O_8_/MoO_2_@MoS_2_‐900, and FeMoOS‐1050 °C, were synthesized. The gas‐sensing response curves of these nanocomposites exposed to 30 ppm NH_3_ at room temperature in 5% relative humidity (RH) are depicted in **Figure** **4**a. The obtained gas‐sensing responses were 59%, 204%, 875%, and 409% for FeMoOS‐600, FeMoOS‐750, Fe_2_Mo_3_O_8_/MoO_2_@MoS_2_‐900, and FeMoOS‐1050 °C, respectively. These results indicated that the Fe_2_Mo_3_O_8_/MoO_2_@MoS_2_‐900 °C nanocomposite exhibited the best gas‐sensing performance. From XRD data, at the annealing temperature over 900 °C, the Fe_2_Mo_3_O_8_ phase disappeared, which is responsible for the decrease in the response. Hence, it can be proposed that the formation of Fe_2_Mo_3_O_8_ is essential for enhancing the gas sensing performance. An optimal annealing temperature of 900 °C was selected for further study.

#### 2.2.2 Gas‐Sensing Performances of the Different Materials

To compare the gas‐sensing performances of different precursors and intermediate materials, gas sensors based on MoS_2_, α‐Fe_2_O_3_/MoS_2_, MoO_2_@MoS_2_, and Fe_2_Mo_3_O_8_/MoO_2_@MoS_2_‐900 °C nanocomposites were constructed and exposed to 30 ppm of NH_3_ at room temperature in 5% RH. Upon exposure to NH_3_, the resistances of all the materials increased, which indicated a typical p‐type semiconductor behavior. The responses 4%, 13%, 143%, and 875% were achieved from MoS_2_, α‐Fe_2_O_3_/MoS_2_, MoO_2_@MoS_2_, and Fe_2_Mo_3_O_8_/MoO_2_@MoS_2_‐900 °C, respectively (Figure 4b). Moreover, the Fe_2_Mo_3_O_8_/MoO_2_@MoS_2_‐900 °C sensor exhibited the fastest response and recovery times of ≈3 min (response) and 60 min (90% recovery) at room temperature in 5% RH. In contrast, MoS_2_ and α‐Fe_2_O_3_/MoS_2_ sensors showed a limited recovery of 30% in 17 min and were unable to recover fully. The MoO_2_@MoS_2_ demonstrated a slightly faster recovery rate of ≈60% within 17 min, although a complete recovery was not achieved. These results indicate that the gas‐sensing response and recovery performance of the original MoS_2_ could be significantly improved by compounding it with α‐Fe_2_O_3_ and annealing in low oxygen partial pressure to form MoO_2_/MoS_2_ heterojunction coupled with Fe_2_Mo_3_O_8_, resulting in a far greater response than the substrate materials.

#### 2.2.3 Dynamic Sensing Performance of Fe_2_Mo_3_O_8_/MoO_2_@MoS_2_‐900 °C Sensor

The temporal dynamic reversible performance of the Fe_2_Mo_3_O_8_/MoO_2_@MoS_2_‐900 °C sensor was investigated by exposing it to 1, 5, 10, 20, 30, 40 and 50 ppm of NH_3_ with the corresponding responses of 57%, 363%, 565%, 728%, 875%, 987%, and 1086%, respectively (Figure 4c). The result suggested that the Fe_2_Mo_3_O_8_/MoO_2_@MoS_2_‐900 °C gas sensor was ultrasensitive and capable of detecting NH_3_ over a wide range of concentrations at room temperature.

A calibration curve of the responses at different NH_3_ concentrations is plotted in Figure 4d. A strong linear relationship between the sensor response and NH_3_ concentration was observed with the correlation coefficient *R^2^* of 0.99, indicating a reliable linear response for NH_3_ concentrations between 1 and 9 ppm. The detection limit (DL) is calculated using the Equation (1):^[^ ^14^ ^]^

(1) DL=3RMS/slope

where RMS represents the root mean squared of noise. The slope was calculated from the linear response between 1 to 9 ppm NH_3_.^[^ ^15^ ^]^ For the Fe_2_Mo_3_O_8_/MoO_2_@MoS_2_‐900 °C sensor, the DL was determined to be 3.7 ppb, which is below the typical ammonia content (1 ppm) found in the exhaled breath of kidney disease patients.^[^ ^9^ ^]^ More importantly, it is also lower than the NH_3_ concentration threshold of 10 ppb, indicating early liver or kidney failure when other symptoms are not shown. Hence, the ultrasensitive Fe_2_Mo_3_O_8_/MoO_2_@MoS_2_‐900 °C sensor can be used not just for domestic monitoring of patients with liver or kidney disease but also for early diagnosis for the prevention of disease.

#### 2.2.4 Selectivity of Fe_2_Mo_3_O_8_/MoO_2_@MoS_2_‐900 °C Sensor

**Figure 5:**
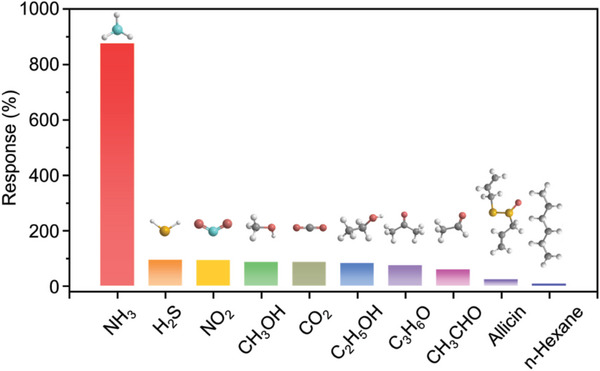
Responses of the Fe_2_Mo_3_O_8_/MoO_2_@MoS_2_‐900 °C sensor to 30 ppm of NH_3_ and interference gases at room temperature and 5% RH.

In practical applications, gas sensors must operate in complex environments where multiple gases are present. Therefore, gas sensors must be highly selective to identify target gases in real environments accurately. The selectivity of the Fe_2_Mo_3_O_8_/MoO_2_@MoS_2_‐900 °C sensor was measured by exposing it to 30 ppm of NH_3_, hydrogen sulfide (H_2_S), nitrogen dioxide (NO_2_), methanol (CH_3_OH), carbon dioxide (CO_2_), ethanol (C_2_H_5_OH), acetone (C_3_H_6_O), acetaldehyde (CH_3_CHO), allicin (which has similar odors to NH_3_) and n‐hexane, at room temperature and 5% RH. The responses were 875%, 93%, 92%, 85%, 85%, 81% 73%, 58%, 22%, and 7%, respectively, as shown in **Figure** **5**. The response to NH_3_ was significantly higher than to the other gases. Such an excellent selectivity toward NH_3_ is critical to the reliability of the sensing result. The relatively low response to other gases might be ascribed to the high activation energy barrier for them on the surface of Fe_2_Mo_3_O_8_/MoO_2_@MoS_2_.^[^ ^13^ ^]^

#### 2.2.5 Repeatability and Long‐Term Stability of Fe_2_Mo_3_O_8_/MoO_2_@MoS_2_‐900 °C Sensor

**Figure 6:**
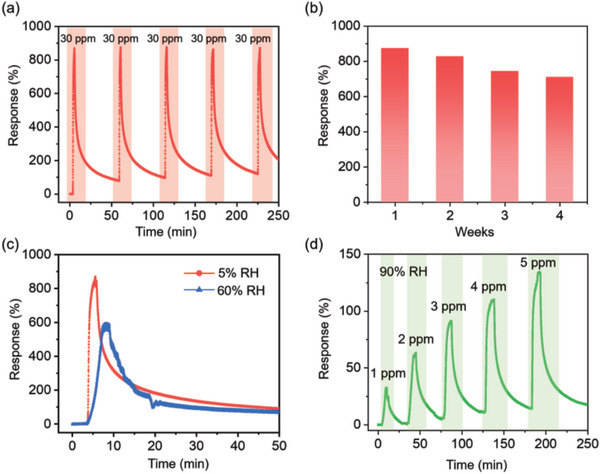
a) Repeatability and b) long‐term stability of Fe_2_Mo_3_O_8_/MoO_2_@MoS_2_‐900 °C sensor. Responses of Fe_2_Mo_3_O_8_/MoO_2_@MoS_2_‐900 °C sensor to c) 30 ppm of NH_3_ at 5% and 60% RH and d) 1–5 ppm of NH_3_ at 90% RH.

The repeatability of the Fe_2_Mo_3_O_8_/MoO_2_@MoS_2_‐900 °C sensor was assessed by subjecting it to five response–recovery cycles of exposure to 30 ppm of NH_3_ at room temperature in 5% RH. As shown in **Figure** **6**a, there is no obvious change in responses after five cycles and remained at ≈875%. However, the recovery gradually slowed down, possibly due to the diffusion of the NH_3_ between the MoS_2_ layers. However, as the response is maintained constant, it can be assumed that the tail of the background and the response peak are contributed by different mechanisms. Further modeling and structural control will be carried out in the future in order to optimize the recovery performance. Nevertheless, the Fe_2_Mo_3_O_8_/MoO_2_@MoS_2_‐900 °C sensor offers excellent repeatability.

The long‐term stability was evaluated by measuring the response of the Fe_2_Mo_3_O_8_/MoO_2_@MoS_2_‐900 °C sensor to 30 ppm of NH_3_ once a week for four consecutive weeks, after each test, keeping the sensor in a dry room temperature air environment (Figure 6b). The results indicated that the response value decreased 19% from 875% to 711% after four weeks. The results further showed that the Fe_2_Mo_3_O_8_/MoO_2_@MoS_2_‐900 °C sensor had remarkable repeatability and long‐term stability for the NH_3_ detection.

#### 2.2.6 Effect of Humidity on Gas‐Sensing Performance of Fe_2_Mo_3_O_8_/MoO_2_@MoS_2_‐900 °C Sensor

Humidity is a dominant component that can interfere with the detection of gases in exhaled human breath and affect the performance of gas sensors. To evaluate this, the performance of the Fe_2_Mo_3_O_8_/MoO_2_@MoS_2_‐900 °C sensor when exposed to 30 ppm of NH_3_ was compared at 5% and 60% RH. The performance was also evaluated at 90% RH by exposing to 1–5 ppm NH_3_. Before introducing NH_3_, the Fe_2_Mo_3_O_8_/MoO_2_@MoS_2_‐900 °C sensor was stabilized to moist air with the desired RH. During the measurement, RH in the gas‐sensing chamber was controlled by passing dry air through a cylindrical water container while introducing dry NH_3_. As shown in Figure 6c, by increasing the RH to 60%, the response to 30 ppm of NH_3_ was decreased to 581% with respect to 875% at 5% RH. At 90% RH, which was close to the RH of exhaled human breath, the responses of 32%, 62%, 90%, 110%, and 134% were achieved by exposing to 1, 2, 3, 4, and 5 ppm of NH_3_, respectively, at room temperature, as shown in Figure 6d.

The above results indicate that the response of the Fe_2_Mo_3_O_8_/MoO_2_@MoS_2_‐900 °C sensor to a specific concentration of NH_3_ decreased with rising RH, although it still preserved a comparatively high response level. At high humidities, water molecules will compete with O_2_ and NH_3_ for their adsorption on the active sites, resulting in hindered NH_3_ oxidation kinetics and reduced sensitivity. Additionally, the adsorbed water could also disrupt the heterostructure and reduce the electron transport ability of the sensor.^[^ ^16^ ^]^

In the realm of 2D material‐based, solid‐state ammonia gas sensors, various strategies such as defect engineering, doping engineering, noble metal modification, and heterojunction structures are usually employed to enhance sensor performance. For instance, the defect‐engineered 2D SnS_2_ sensor with S vacancies exhibited a response of 420% to 500 ppm NH_3_ at room temperature with a detection limit of 20 ppm.^[^ ^25^ ^]^ NO_2_‐doped graphene sensor, representing doping engineering, showed a response of 14% to 30 ppm NH_3_ at room temperature with a relatively low DL of 200 ppb.^[^ ^26^ ^]^ For noble metal modification, the Pt‐MoS_2_ sensor demonstrated a response of 36% to 70 ppm NH_3_ at room temperature, with a lower DL of 130 ppb.^[^ ^27^ ^]^ With a designed heterojunction, the MoS_2_/ZnO sensor delivered a response of 70% to 30 ppm NH_3_, with a poor DL of 920 ppb.^[^ ^28^ ^]^ Additionally, the GaN/rGO sensor shows a response of 92% to 200 ppm NH_3_, with a DL of 28 ppb.^[^ ^29^ ^]^ More sensor performance comparisons can be found in **Table** **1**. In our work, the Fe_2_Mo_3_O_8_/MoO_2_@MoS_2_‐900 °C sensor combines the advantages of defect engineering through O doping in MoS_2_ and a heterojunction structure by forming Fe_2_Mo_3_O_8_ on MoO_2_@MoS_2_. To our knowledge, it outperformed most of the NH_3_ sensors in the literature based on a range of technologies, as we summarized here, with an outstanding response of 875% to 30 ppm NH_3_ at a superior DL of 3.7 ppb. Notably, the Fe_2_Mo_3_O_8_/MoO_2_@MoS_2_‐900 °C sensor exhibits exceptional sensitivity, selectivity, and broad dynamic range, which underscore its potential for clinical diagnosis of liver and kidney disease at an early stage. Compared with the literature, our Fe_2_Mo_3_O_8_/MoO_2_@MoS_2_‐900 °C sensor is the only device offering a detection limit below the threshold of 10 ppb NH_3_ found in the breath of patients who might be suffering from liver or kidney failure.^[^ ^2^ ^]^

**Table 1:** Responses of different materials to NH_3_ gas at room temperature.

Material	NH_3_ Concentration [ppm]					DL [ppb]	Ref.
	1	5	10	20	30		
	Response [%]						
CeO_2_	200	250	350	–	500	500	[17]
Ti_3_C_2_ Mxene	–	–	0.62	–	–	10 000	[18]
Graphene	–	–	–	13	–	27	[19]
Ti_3_C_2_Tx/WO_3_	22	–	–	–	–	1000	[20]
MoS_2_/MoO_3_	15	–	24	30	39	1000	[21]
MoS_2_/Co_3_O_4_	26	64	–	–	–	100	[11]
NiO/MoS_2_	31	45	63	80	90	250	[22]
MoS_2_/CuO	–	8	–	–	–	5000	[23]
MoS_2_/ZnO	18	25	37	–	–	12	[24]
2D SnS_2_	–	–	–	150	–	20 000	[25]
NO_2_‐doped graphene	–	–	–	–	14	200	[26]
Pt‐MoS_2_	–	17	–	–	24	130	[27]
MoS_2_/ZnO	–	9	21	35	70	920	[28]
GaN/rGO	23	42	–	60	–	28	[29]
Fe_2_Mo_3_O_8_/MoO_2_@MoS_2_‐900 °C	57	363	565	728	875	3.7	This work

“‐” The response was not given in the literature.

#### 2.2.7 Detection of NH_3_ in the Exhaled Breath of Kidney Disease Patients by Fe_2_Mo_3_O_8_/MoO_2_@MoS_2_‐900 °C Sensor

**Figure 7:**
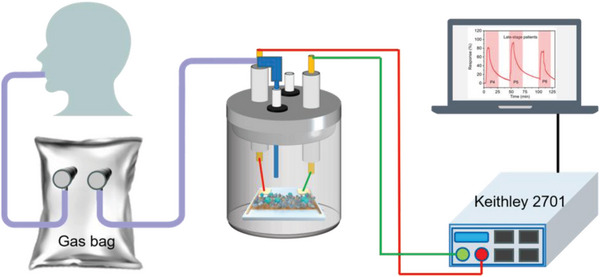
Schematic diagram of human exhaled breath collection and gas‐sensing test.

To evaluate the NH_3_ sensor's performance in the diagnosis of kidney disease, the NH_3_ concentration in the exhaled breath was measured using the Fe_2_Mo_3_O_8_/MoO_2_@MoS_2_‐900 °C sensor. In this experiment, internationally recognized definitions for the stages of chronic kidney disease (CKD) were adopted. Early‐stage and late‐stage kidney disease patients are referred to the CKD1‐2 and CKD5 stages, respectively. With patients’ consent, the oral exhaled breath was sampled from three healthy people (H1, H2, H3), three early‐stage kidney disease patients (P1, P2, and P3), and three late‐stage kidney disease patients (P4, P5, and P6) using medical grade Tedlar gas bags before their morning tooth brushing routine. The RH of these collected human exhaled gas samples was measured to be approximately 90% using a hygrometer. The Fe_2_Mo_3_O_8_/MoO_2_@MoS_2_‐900 °C sensor was preconditioned in a 90% RH air environment. Once the resistance of the sensor was stabilized, the exhaled breath samples were introduced. To measure the NH_3_ concentration using a homemade gas‐sensing test station,^[^ ^30^, ^31^ ^]^ as depicted in **Figure** **7**.

**Figure 8:**
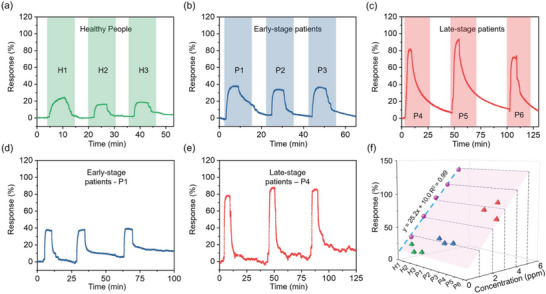
Application of the Fe_2_Mo_3_O_8_/MoO_2_@MoS_2_‐900 °C sensor into exhaled breath detection: Responses to the exhaled breath of a) three healthy people (H1, H2, and H3), b) three early‐stage patients (P1, P2, and P3) and c) three late‐stage patients (P4, P5, and P6). Repeatability of the response to the exhaled breath of d) P1 and e) P4. f) Linear fit of the responses to NH_3_ concentrations of 1–5 ppm at room temperature and 90% RH, and the responses to the exhaled breath of three healthy people and six patients.

The expiratory responses of three healthy people, H1, H2, and H3, were relatively low at 24%, 16%, and 18%, respectively, as illustrated in **Figure** **8**a. Subsequently, we examined the ability of the Fe_2_Mo_3_O_8_/MoO_2_@MoS_2_‐900 °C sensor to distinguish between patients with early‐ and late‐stage kidney disease. Figure 8b shows slightly higher responses from three early‐stage patients (P1, P2, and P3) of 38%, 34%, and 36%. Figure 8c presents the response values from three late‐stage patients (P4, P5, and P6) of 81%, 92%, and 72%. The reliability of the reading is demonstrated by the repeated measurements from patient P1 (early‐stage) and patient P4 (late‐stage) three times. Figure 8d,e indicated that the responses for P1 were 38%, 38%, and 40%, while P4 showed responses of 78%, 87%, and 85%. The relative standard deviations are 2.99% and 5.67% for patients P1 and P4, respectively, indicating good consistency and reliability. Significant differentiation in the exhaled breath responses between two groups of patients at CKD1‐2 and CKD5 stages is distinctively observed. Hence, the sensitivity and the dynamic range of the Fe_2_Mo_3_O_8_/MoO_2_@MoS_2_‐900 °C sensor are sufficient for distinguishing the different stages of kidney disease, which has the potential for clinical application.

Furthermore, a calibration curve for the sensor's response to NH_3_ concentrations ranging from 1–5 ppm at 90% RH was established and presented in Figure 8f. The high correlation coefficient (*R^2^* = 0.99) confirmed a reliable linear relationship, which is important for the accuracy of measurements. This calibration curve was then utilized to determine the NH_3_ concentration in the exhaled breath of kidney disease patients. For healthy people H1‐H3 and patients P1–P6, the NH_3_ concentrations were determined to be 0.56, 0.24, 0.32, 1.11, 0.95, 1.03, 2.82, 3.25, and 2.46 ppm, aligning with previously reported values in literature.^[^ ^7^ ^]^ Therefore, the Fe_2_Mo_3_O_8_/MoO_2_@MoS_2_‐900 °C sensor has been demonstrated as an affordable but reliable diagnosis device to monitor kidney disease by measuring the concentration of NH_3_ in the exhaled breath.

#### 2.2.8 Sensing Mechanism

During the test, it was observed that MoO_2_@MoS_2_, Fe_2_Mo_3_O_8,_ and Fe_2_Mo_3_O_8_/MoO_2_@MoS_2_‐900 °C nanocomposites all showed *p*‐type gas‐sensing behavior with holes as the major charge carriers. MoO_2_@MoS_2_ is the main conductive component in the nanocomposite, while Fe_2_Mo_3_O_8_ offers active catalytic sites for the adsorption and reaction of the NH_3_ molecule. The band gaps of MoO_2_@MoS_2_ and Fe_2_Mo_3_O_8_ were determined as 2.23 and 1.14 eV (Figure S3a, Supporting Information) from the UV–vis absorption measurements. The work functions, *W*_f_, of MoO_2_@MoS_2_ and Fe_2_Mo_3_O_8_ were identified to be 5.22 and 6.92 eV, using the UV photoelectron spectroscopy and Equation (2):^[^ ^32^ ^]^

(2) $$W_{f}=hv-\left(E_{\mathrm{Cutoff}}-E_{\mathrm{Fermi}}\right)$$

Here, *hv* is the ultraviolet photon energy (21.22 eV), *E*_Cutoff_ represents the secondary electron cutoff edge, and *E*_Fermi_ represents the fermi edge (Figure S3b, Supporting Information). At the heterojunction between the MoO_2_@MoS_2_ and Fe_2_Mo_3_O_8_, electrons are expected to be transferred from MoO_2_@MoS_2_ to Fe_2_Mo_3_O_8_ due to the smaller work function of MoO_2_@MoS_2_, resulting in a hole depletion layer on the surface of p‐type Fe_2_Mo_3_O_8_. When the gas sensor is exposed to air, O_2_ can easily adsorbed on the surface of Fe_2_Mo_3_O_8_, which is electron‐rich. During this process, oxygen molecules acted as electron acceptors. The O_2_ adsorption increases the carrier (hole) concentration in the p‐type Fe_2_Mo_3_O_8_, which effectively reduces the thickness of the hole depletion layer. Thus, the resistance of the nanocomposite is decreased. When NH_3_ is adsorbed, it reacts with $O_{2}^{-}$ to generate NO and H_2_O, while the Fe_2_Mo_3_O_8_ will gain electrons. Consequently, the majority carrier (hole) concentration in the composite decreases, leading to an increase in the thickness of the depletion layer and the resistance of the composite material.

**Figure 9:**
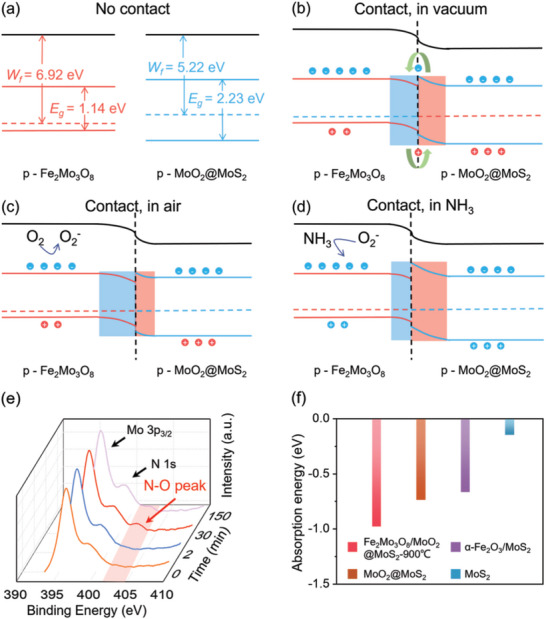
The diagram of the energy band of Fe_2_Mo_3_O_8_/MoO_2_@MoS_2_‐900 °C: a) no contact between the materials, b) contact between the materials in a vacuum, c) contact between the materials in air, and d) contact between the materials in NH_3_. XPS analysis and theoretical calculation based on DFT: e) XPS spectra of N─O peak, f) the adsorption energy of NH_3_.

Energy band diagrams of the Fe_2_Mo_3_O_8_/MoO_2_@MoS_2_‐900 °C composite when exposed to different gases are shown in **Figure** **9**a–d. The sequence of the surface reactions could be described in the following:^[^ ^11^ ^]^

(3) $$O_{2(\mathrm{gas})}\to O_{2(\mathrm{abs})}$$

(4) $$O_{2(\mathrm{abs})}+e^{-}\to O_{2(\mathrm{abs})}^{-}$$

(5) $$NH_{3(\mathrm{gas})}\to NH_{3(\mathrm{abs})}$$

(6) $$4NH_{3(\mathrm{abs})}+5O_{2(\mathrm{abs})}^{-}\to 4\mathrm{NO}+6H_{2}O+5e^{-}$$

To verify the reaction process, XPS analysis was conducted on the Fe_2_Mo_3_O_8_/MoO_2_@MoS_2_‐900 °C sensor after exposure to NH_3_ gas for 0, 2, and 30 min and then recovered for 2 h (Figure 9e). The analysis revealed no significant changes in the XPS signals from Mo 3d, O 1s, and S 2p. However, a new, albeit weak, peak at 402.6 eV associated with the N 1s peak was detected in the sample exposed to NH_3_ for 30 min, indicating the formation of N─O bonds and the presence of NO moiety.^[^ ^33^ ^]^ The intensity of the N─O peak decreased after a 2‐h recovery period, indicating that the adsorption of the reaction product is reduced during the recovery process at room temperature. Thus, the desorption energy barrier must be at a similar scale as the thermal energy at room temperature. Such property is essential for the reproducibility of the response to NH_3_ at room temperature, which is essential for the sensor to be operated under ambient conditions. The XPS results confirmed that NO is the reaction product for the NH_3_ catalytically oxidation.

Furthermore, to investigate the influence of Fe_2_Mo_3_O_8_ on the gas‐sensing response of composite materials, DFT was conducted to determine the adsorption energy of NH_3_ on various materials and the corresponding change in free energy during NH_3_ oxidation. In Figure 9f, the maximum adsorption energies of NH_3_ on Fe_2_Mo_3_O_8_/MoO_2_@MoS_2_‐900 °C, MoO_2_/MoS_2_, α‐Fe_2_O_3_/MoS_2_ and MoS_2_ were −0.98, −0.74, −0.67 and −0.15 eV, respectively. The negative values of the adsorption energies indicated an exothermic adsorption process. Consequently, NH_3_ adsorption was most favorable on Fe_2_Mo_3_O_8_/MoO_2_@MoS_2_‐900 °C, suggesting that it facilitated the subsequent NH_3_ oxidation reaction.

**Figure 10:**
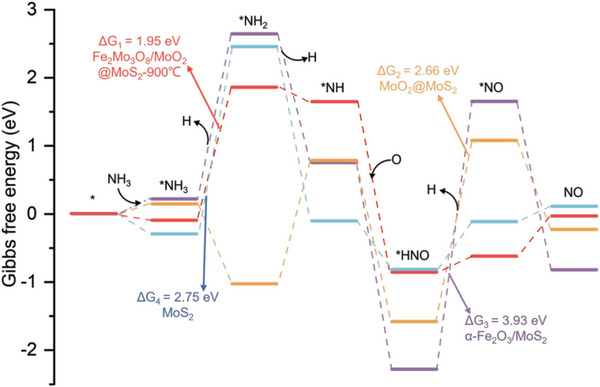
The free energy changes during NH_3_ oxidation reaction with Fe_2_Mo_3_O_8_/MoO_2_@MoS_2_‐900 °C, MoO_2_/MoS_2_, α‐Fe_2_O_3_/MoS_2_ and MoS_2_.

The oxidation reaction mechanism and the associated free energy change (ΔG) of NH_3_ across four catalysts, including Fe_2_Mo_3_O_8_/MoO_2_@MoS_2_‐900 °C, MoO_2_/MoS_2_, α‐Fe_2_O_3_/MoS_2_ and MoS_2_ were illustrated in **Figure** **10**. The reaction involves six steps with corresponding intermediates. 1) NH_3_ initially adsorbs on the active site of the catalyst to form *NH_3_. 2) NH_3_ is partially dehydrogenated to generate *NH_2_. 3) *NH_2_ is further dehydrogenated to form *NH. 4) *NH reacts with O to produce *HNO. 5) *HNO releases an H atom, resulting in the formation of *NO. 6) Finally, *NO desorbs to release gaseous NO and recover the active site. For Fe_2_Mo_3_O_8_/MoO_2_@MoS_2_‐900 °C and MoS_2_, the rate‐limiting steps are determined by the calculation of the energy barriers. On the Fe_2_Mo_3_O_8_/MoO_2_@MoS_2_‐900 °C and MoS_2_, the rate‐limiting steps are the first dehydrogenation with the ΔG values of 1.95 eV and 2.75 eV, respectively. On the MoO_2_@MoS_2_ and α‐Fe_2_O_3_/MoS_2_, the rate‐limiting steps involve the deprotonation of HNO, exhibiting ΔG values of 2.66 and 3.93 eV, respectively. It was noteworthy that Fe_2_Mo_3_O_8_/MoO_2_@MoS_2_‐900 °C shows the lowest ΔG at the rate‐controlling step, indicating its superior catalytic efficiency in NH_3_ oxidation. The result suggested that Fe_2_Mo_3_O_8_ forms a heterogeneous interface with MoO_2_@MoS_2_ to facilitate electron transfer and activate the adsorbed O_2_, leading to favorable NH_3_ adsorption and catalytic oxidation at room temperature. Such a unique interface structure guarantees a high response and very low detection limit for NH_3_, which is required for the diagnosis of early‐stage kidney disease.

## 3 Conclusion

In summary, Fe_2_Mo_3_O_8_/MoO_2_@MoS_2_‐900 °C nanocomposite was successfully synthesized and optimized for the room‐temperature NH_3_ gas sensor with potential application in noninvasive diagnosis of different stages of kidney disease. The sensor exhibited an extremely high response with an ultra‐low detection limit of 3.7 ppb for NH_3_. Through analyzing exhaled gases, the Fe_2_Mo_3_O_8_/MoO_2_@MoS_2_‐900 °C sensor demonstrated a unique ability to differentiate between early‐ and late‐stage kidney disease in patients. Moreover, the gas‐sensing mechanism and associated intermediates were elucidated by XPS detections and DFT calculations. The excellent gas‐sensing performance of the sensor was established due to the formation of heterojunctions between MoS_2_, MoO_2_, and Fe_2_Mo_3_O_8_ and the exceptional NH_3_ adsorption and catalytic oxidation activity of the Fe_2_Mo_3_O_8_. The remarkable capabilities of the Fe_2_Mo_3_O_8_/MoO_2_@MoS_2_‐900 °C sensor offer a viable method for analyzing exhaled gases in individuals with kidney disease, suggesting a novel strategy for early diagnosis and management of kidney disease.

## 4 Experimental Section

### Chemical Reagents

Ferric chloride hexahydrate (FeCl_3_•6H_2_O, analytical research (AR) grade), sodium molybdate dihydrate (Na_2_MoO_4_•2H_2_O, AR grade), and thiourea (CH_4_N_2_S, AR grade) were purchased from Shanghai Macklin Biochemical Co., Ltd, China. Citric acid monohydrate (C_6_H_8_O_7_•H_2_O, AR grade) and deionized water (H_2_O) were purchased from Shanghai Sinopharm Chemical Reagent Co., Ltd., China. Ethyl alcohol (C_2_H_5_OH, AR grade) was purchased from Zhenxing No.1 Chemical Plant, China.

### Synthesis of Sensing Materials

A typical procedure was used to synthesize MoS_2_. First, 484 mg of Na_2_MoO_4_•2H_2_O and 684 mg of CH_4_N_2_S were mixed and dissolved in 70 mL of deionized water, and the mixture was stirred for 30 min. Then, 462 mg of C_6_H_8_O_7_•H_2_O was added to the solution and stirred for an additional 10 min. The resulting solution was transferred into a 100 mL autoclave and heated at 200 °C for 21 h. Finally, the mixture was washed three times with deionized water and anhydrous ethanol using centrifugation, and heated at 60 °C for 12 h.

To synthesize the α‐Fe_2_O_3_/MoS_2_ complex, 460 mg of FeCl_3_•6H_2_O was dissolved in 60 mL deionized water and stirred for 30 min. Then, 140 mg of MoS_2_ was added to the solution, which was stirred for another 20 min. The solution was transferred into a 100 mL autoclave and heated at 180 °C for 12 h.

To synthesize the FeMoOS‐600, FeMoOS‐750, FeMoOS‐1050, and Fe_2_Mo_3_O_8_/MoO_2_@MoS_2_‐900 °C, α‐Fe_2_O_3_/MoS_2_ composite was annealed at different temperatures,100 mg samples of α‐Fe_2_O_3_/MoS_2_ were evenly spread on small porcelain boats and placed in a tube furnace. The samples were then annealed at 600, 750, 900, and 1050 °C in a low oxygen partial pressure atmosphere.

### Material Characterization

The morphologies of the sensing materials were examined using SEM (S‐4800N, Hitachi, Japan) and TEM (JEM‐2100F, JEOL, Japan). The crystallinity was investigated using XRD (D8 ADVANCE, Bruker, Cu kα, 380 eV, Germany). The composition was determined via XPS (ESCAlab250, ThermoFisher, Al X‐ray source, 1486.6 eV, USA). The concentration of Fe in the nanocomposite was determined by glow discharge mass spectrometry (GD‐MS) (Auto Concept GD 90, Mass Spectrometry Instruments Co., Ltd., UK). The band gap and work function of the materials were measured by UV‐visible spectroscopy (U‐4100, Hitachi, Japan) and UV photoelectron spectroscopy (250Xi, ThermoFisher, He I line, 21.22 eV, USA), respectively.

### Gas Sensor Fabrication

The Fe_2_Mo_3_O_8_/MoO_2_@MoS_2_‐900 °C sensor was fabricated on interdigital electrodes composed of Fe_2_Mo_3_O_8_/MoO_2_@MoS_2_‐900 °C sensing materials and interdigital electrodes, as well as for the other sensors based on MoO_2_@MoS_2_, α‐Fe_2_O_3_/MoS_2_, MoS_2_, FeMoOS‐600, FeMoOS‐750, and FeMoOS‐1050 °C materials. The interdigital electrode is 1 × 1 × 0.05 cm^3^ in size and composed of an Al_2_O_3_ ceramic substrate and Au electrode on it. The interdigital electrodes were cleaned under sonication with acetone, deionized water, and ethanol before use. Then, the annealed Fe_2_Mo_3_O_8_/MoO_2_@MoS_2_‐900 °C suspension was carefully dripped onto the surface of the electrode. The electrode was then placed back into the oven at 60 °C for 6 h to form a gas sensor based on the Fe_2_Mo_3_O_8_/MoO_2_@MoS_2_‐900 °C sensing material.

### Gas‐Sensing Response Measurement

To investigate the gas‐sensing capabilities, a dedicated gas‐sensing apparatus was constructed with a gas cylinder, a plastic conduit, a gas‐sensing chamber, and a resistance acquisition device, as shown in Figure 7. The cylinders were filled with dry air and dry NH_3_ respectively. Within the gas‐sensing chamber, there was a cylindrical cavity with a volume of 300 mL and several venting orifices. The interdigital electrode loaded with the Fe_2_Mo_3_O_8_/MoO_2_@MoS_2_‐900 °C nanocomposite was fixed in the cylindrical cavity using a platinum electrode clamp. The bubbling method was applied to adjust the moisture of the injected gas. Typically, NH_3_ with different relative humidity was obtained by mixing the wet air with dry NH_3_, and all the gas flow ratios were regulated by mass flow controllers. The final gas flow rate was fixed at 600 mL min^−1^. The change in the resistance of the sensor was measured by the Keithley 2701 resistance acquisition device. The sensor response was calculated using the equation Response (*R*) = |*Rg* – *Ra*| / *Ra* × 100%, where *Rg* represented the sensor resistance in the presence of NH_3_, and *Ra* represented the sensor resistance in air. The gas‐sensing experiments were conducted at room temperature and 5% RH, if without any special indication.

In the exhaled breath test experiments of kidney disease patients, informed consent was given freely and voluntarily by all participants after receiving comprehensive information and explanations. The study was reviewed and approved by the Medical Ethics Committee of Shanghai Pudong New Area People's Hospital, with the approval number K63. The exhaled breath was collected in the mouth using a Tedlar bag (Dupont USA), which is constructed from polyvinyl fluoride film with a film thickness of ≈0.05 mm. The gas sample collection procedure involves opening the gas outlet of the gas sampling bag, positioning the volunteer's mouth toward the outlet, and exhaling slowly for a duration exceeding 20 s. Subsequently, the gas outlet is sealed to conclude the collection process.

### Theoretical Calculation

DFT was used as implemented in the Vienna Ab initio simulation package (VASP) for all calculations. The exchange‐correlation potential is described by using the generalized gradient approximation of Perdew‐Burke‐Ernzerhof. The projector augmented‐wave method is employed to treat interactions between ion cores and valence electrons. The plane‐wave cutoff energy was fixed to 400 eV. Given structural models were relaxed until the Hellmann‐Feynman forces were <−0.01 eV Å^−1^ and the change in energy <10^−5^ eV was attained. The basic structure of the Fe_2_Mo_3_O_8_/MoO_2_@MoS_2_‐900 °C composite for DFT calculation consists of three layers of distinct compounds arranged in a heterostructure. The bottom layer comprises MoS_2_ with the (002) plane serving as the outer crystal plane, the middle layer is MoO_2_ with the (−111) plane as the outer crystal plane, and the top layer consists of Fe_2_Mo_3_O_8_ with the (102) plane as the outer crystal plane. This composite material is situated within a vacuum layer with a thickness of ≈3.5 nm. The adsorption energy (*E*_ads_) was calculated as:

(7) $$E_{\mathrm{ads}}=E\left(\mathrm{system}\right)-E\left(\mathrm{catalyst}\right)-E\left(\mathrm{species}\right)$$

where *E*(system), *E*(catalyst), and *E*(species) are the total energy of the optimized system with adsorbed species, the isolated catalyst, and species, respectively.

The Gibbs free energy change is defined as:

(8) $$\Delta G=\Delta E\;+\;\Delta E_{\mathrm{ZPE}}-T\Delta S$$

where Δ*E* is the electronic energy calculated with VASP, Δ*E*_ZPE_, and Δ*S* are the zero‐point energy difference and the entropy change between the products and reactants, respectively, and *T* is the temperature (298.15 K).

## Conflict of Interest

The authors declare no conflict of interest.

## Supporting information

Supporting Information

## Data Availability

The data that support the findings of this study are available from the corresponding author upon reasonable request.
