# Supplementary material for: Highly Sensitive Room‐Temperature Detection of Ammonia in the Breath of Kidney Disease Patients Using Fe2Mo3O8/MoO2@MoS2 Nanocomposite Gas Sensor
Source: Adv Sci (Weinh). 2024 Jul 3;11(32):2405942. doi: 10.1002/advs.202405942 (PMC11347992; doi:10.1002/advs.202405942)

Supporting Information

Highly Sensitive Room-Temperature Detection of Ammonia in the Breath of Kidney Disease Patients Using Fe_2_Mo_3_O_8_/MoO_2_@MoS_2_ Nanocomposite Gas Sensor

*Xian Li, Wang Zeng, Shangjun Zhuo, Bangwei Qian, Qiao Chen, Qun Luo, Rong Qian**

**
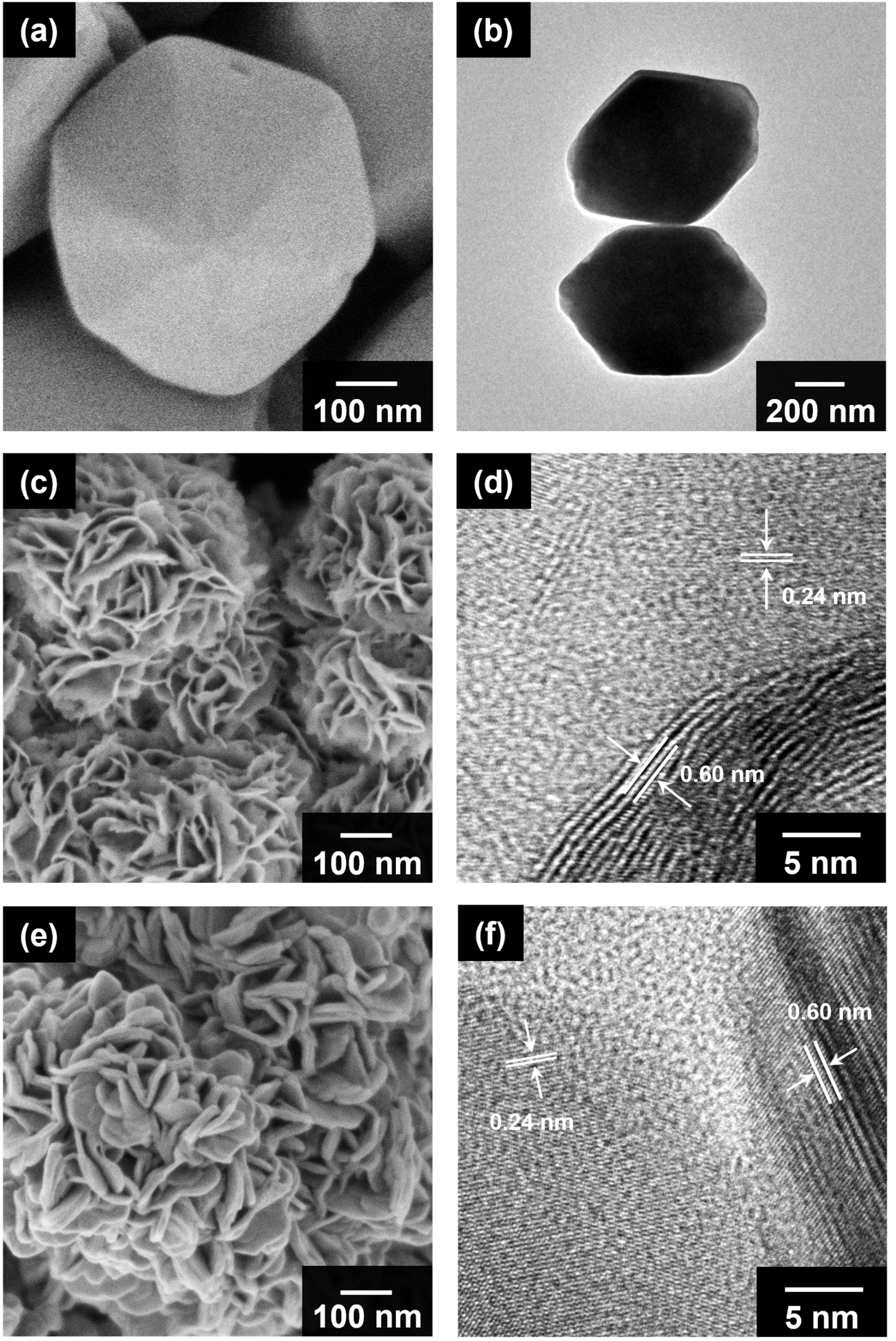
**

**Figure S1.** SEM and TEM images of (a, b) α-Fe_2_O_3_, (c, d) MoS_2_, (e, f) MoO@MoS_2_.

**
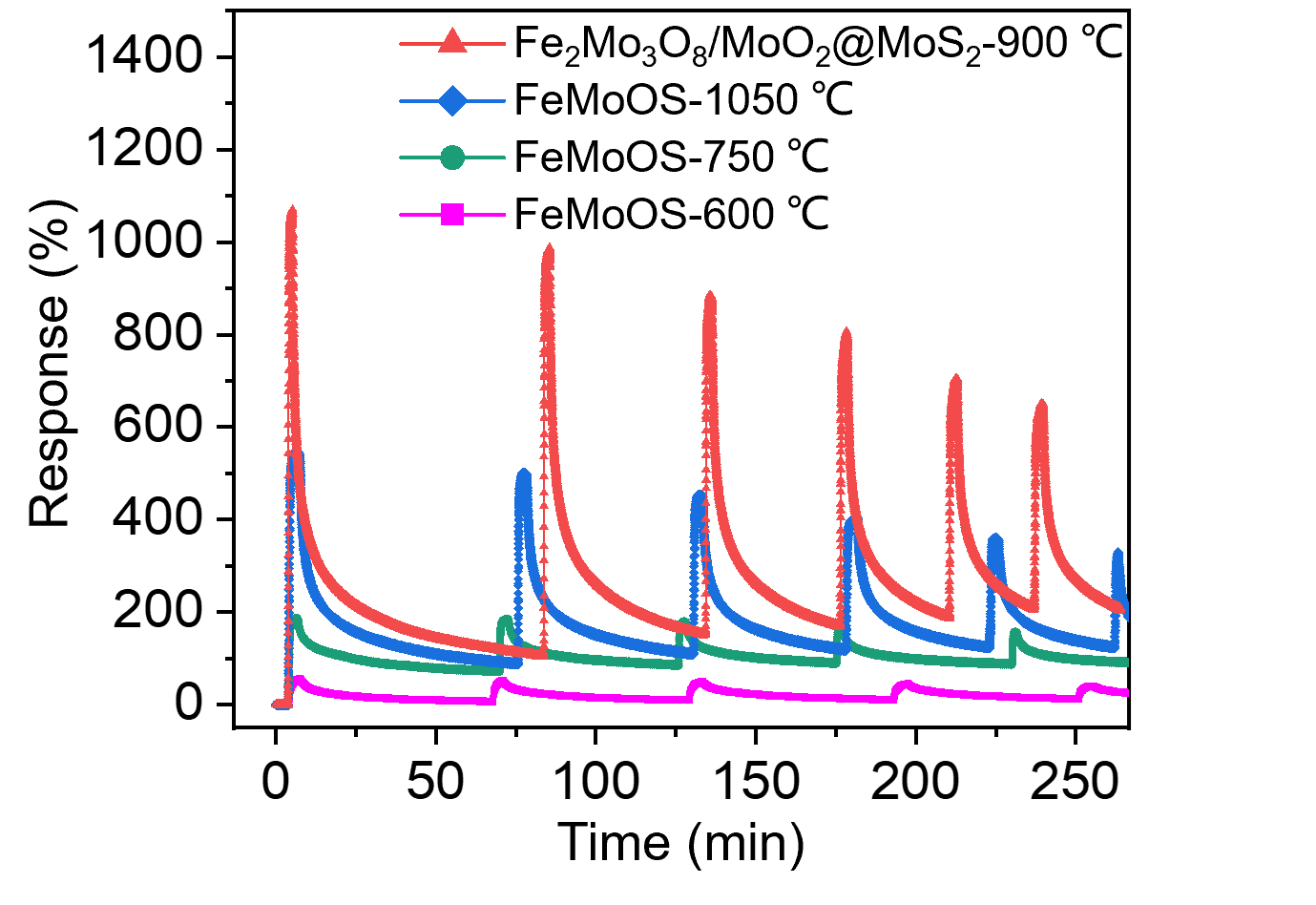
**

**Figure S2.** Gas-sensing sensitivity of Fe_2_Mo_3_O_8_/MoO_2_@MoS_2_-900°C, FeMoOS-1050°C, FeMoOS-750°C and FeMoOS-600°C for NH_3_ at concentrations of 5–50 ppm at room-temperature and 5% RH.

**
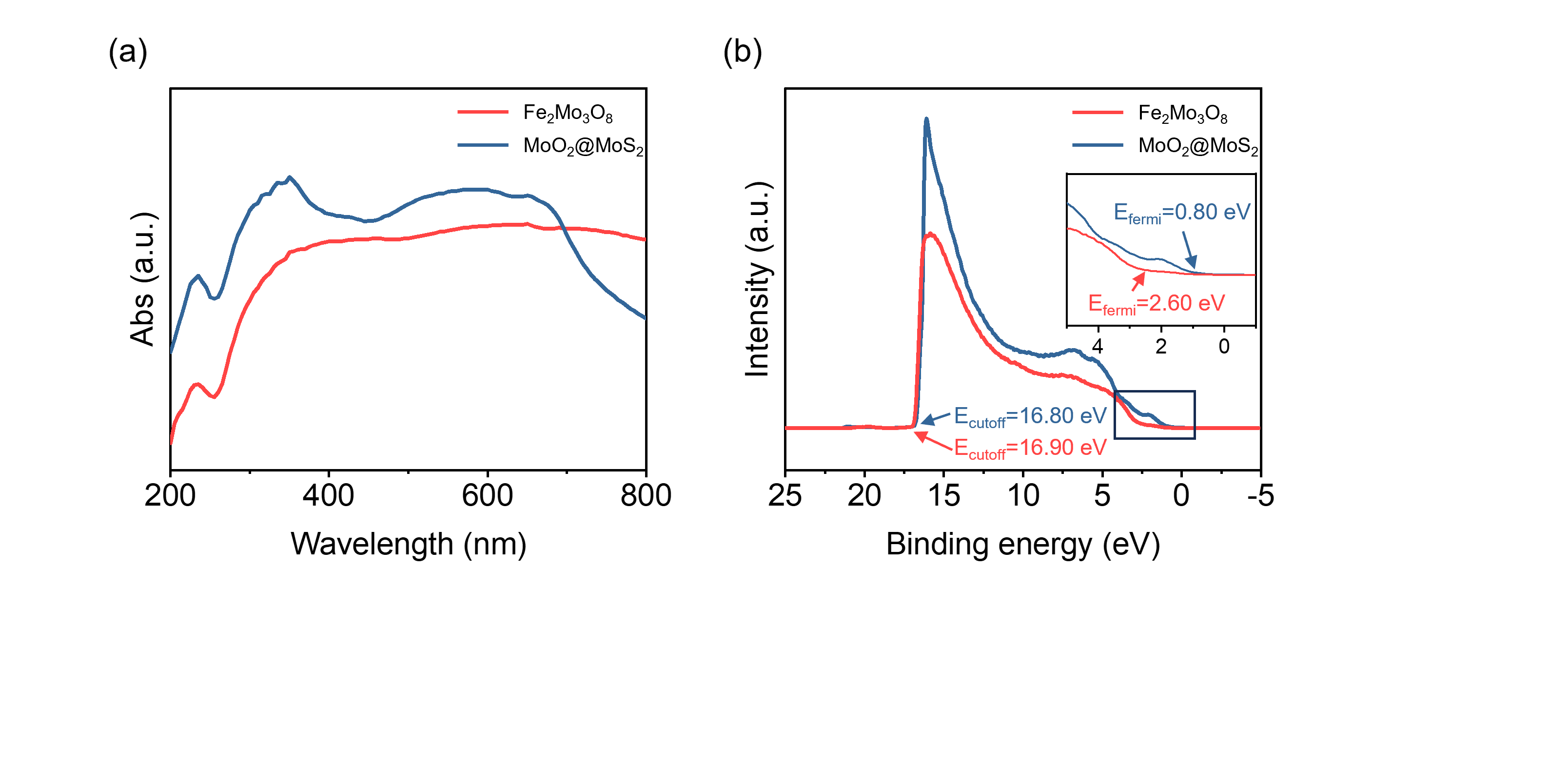
**

**Figure S3.** (a) UV-Vis spectra of Fe_2_Mo_3_O_8_ and MoO_2_@MoS_2_. (b) UPS of Fe_2_Mo_3_O_8_ and MoO_2_@MoS_2_.

**
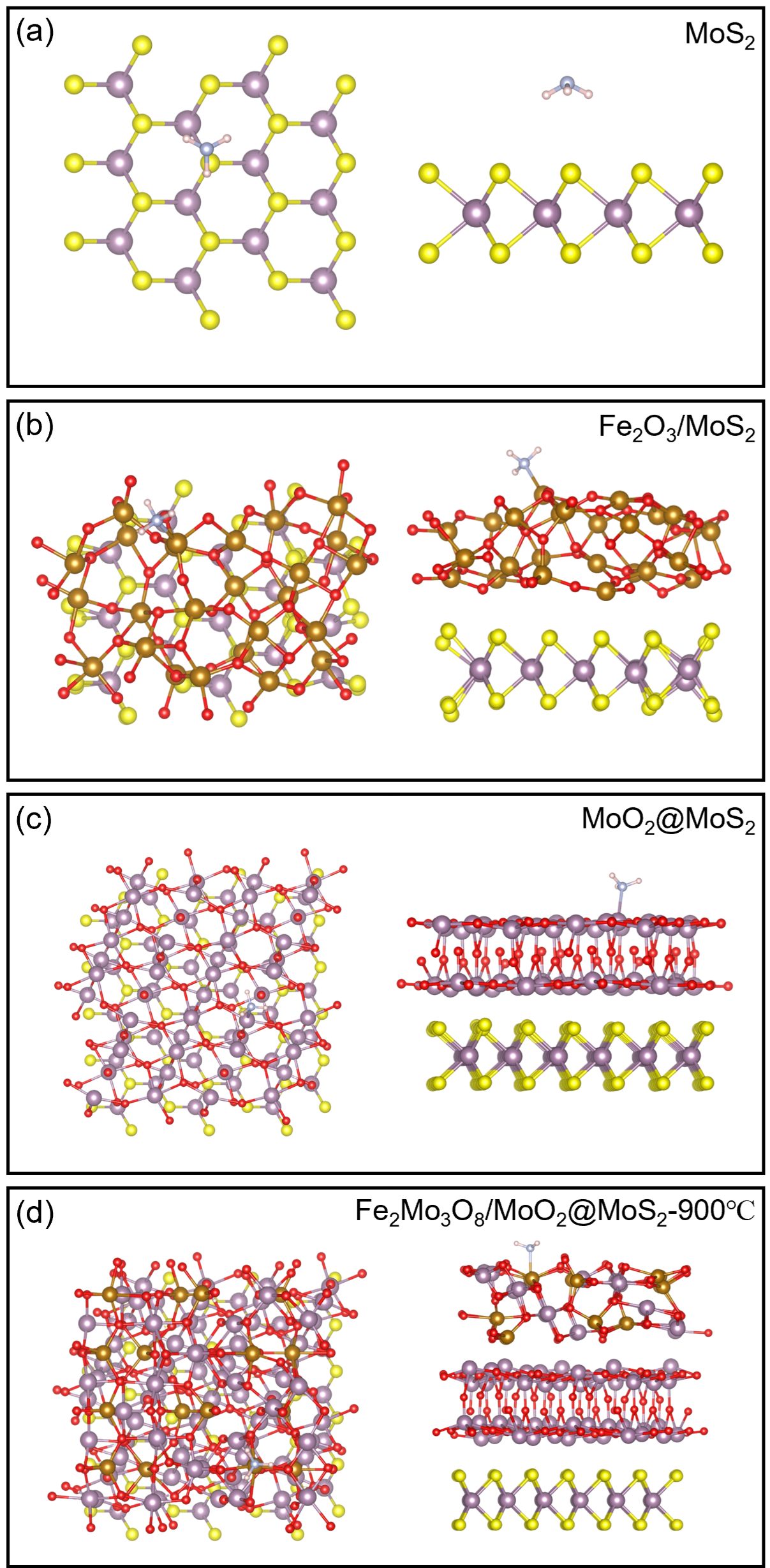
**

**Figure S4.** DFT calculation, NH_3_ adsorption configurations on the nanocomposites: NH_3_ adsorption configurations on (a) MoS_2_, (b) Fe_2_O_3_/MoS_2_, (c) MoO_2_@MoS_2_ and (d) Fe_2_Mo_3_O_8_/MoO_2_@MoS_2_-900°C.

**Table of Contents**

An ultrasensitive NH_3_ gas sensor is developed based on Fe_2_Mo_3_O_8_/MoO_2_@MoS_2_-900°C nanocomposite. The sensor effectively distinguishes between patients with early- and late-stage kidney disease by quantitative analysis of NH_3_ in their exhaled breath. This novel approach offers a promising method for the early diagnosis and management of kidney disease.

Xian Li, Wang Zeng, Shangjun Zhuo, Bangwei Qian, Qiao Chen, Qun Luo, Rong Qian*

**Highly Sensitive Room-Temperature Detection of Ammonia in the Breath of Kidney Disease Patients Using Fe_2_Mo_3_O_8_/MoO_2_@MoS_2_ Nanocomposite Gas Sensor**


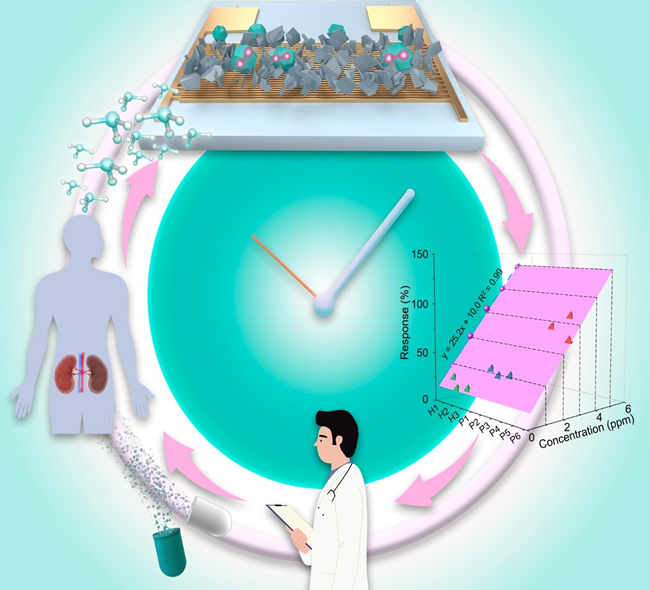

Supplement: Supplementary file 1 — Supporting Information [file ADVS-11-2405942-s001.docx]
